# GluN1 and GluN2A NMDA Receptor Subunits Increase in the Hippocampus during Memory Consolidation in the Rat

**DOI:** 10.3389/fnbeh.2016.00242

**Published:** 2017-01-13

**Authors:** Magali C. Cercato, Cecilia A. Vázquez, Edgar Kornisiuk, Alejandra I. Aguirre, Natalia Colettis, Marina Snitcofsky, Diana A. Jerusalinsky, María V. Baez

**Affiliations:** ^1^Laboratorio de Neuroplasticidad y Neurotoxinas, Instituto de Biología Celular y Neurociencia, Universidad de Buenos Aires (UBA)-CONICETBuenos Aires, Argentina; ^2^Ciclo Básico Común-Universidad de Buenos AiresBuenos Aires, Argentina; ^3^1UA de Biología Celular, Histología, Embriología y Genética, Departamento de Histología, Facultad de Medicina, Universidad de Buenos AiresBuenos Aires, Argentina

**Keywords:** LTM, hippocampus, NMDAR, GluN1, GluN2A, synaptic plasticity

## Abstract

It is widely accepted that NMDA receptors (NMDAR) are required for learning and memory formation, and for synaptic plasticity induction. We have previously shown that hippocampal GluN1 and GluN2A NMDAR subunits significantly increased following habituation of rats to an open field (OF), while GluN2B remained unchanged. Similar results were obtained after CA1-long-term potentiation (LTP) induction in rat hippocampal slices. Other studies have also shown NMDAR up regulation at earlier and later time points after LTP induction or learning acquisition. In this work, we have studied NMDAR subunits levels in the hippocampus and prefrontal cortex (PFC) after OF habituation and after object recognition (OR), to find out whether rising of NMDAR subunits is a general and structure-specific feature during memory formation. In 1, 2 and 3 month old rats there was an increase in hippocampal GluN1 and GluN2A, but not in GluN2B levels 70 min after OF habituation. This rise overlaps with early phase of memory consolidation, suggesting a putative relationship between them. The increases fell down to control levels 90 min after training. Similar results were obtained in the hippocampus of adult rats 70 min after OR training, without changes in PFC. Following OF test or OR discrimination phase, NMDAR subunits remained unchanged. Hence, rising of hippocampal GluN1 and GluN2A appears to be a general feature after novel “spatial/discrimination” memory acquisition. To start investigating the dynamics and possible mechanisms of these changes, we have studied hippocampal neuron cultures stimulated by KCl to induce plasticity. GluN1 and GluN2A increased both in dendrites and neuronal bodies, reaching a maximum 75 min later and returning to control levels at 90 min. Translation and/or transcription and mobilization differentially contribute to this rise in subunits in bodies and dendrites. Our results showed that the NMDAR subunits increase follows a similar time course both *in vitro* and *in vivo*. These changes happen in the hippocampus where a spatial representation of the environment is being formed making possible short term and long term memories (STM and LTM); appear to be structure-specific; are preserved along life; and could be related to synaptic tagging and/or to memory consolidation of new spatial/discrimination information.

## Introduction

Memories are internal representations of previously learnt experiences. Depending on their lasting, memories are currently classified as short term and long term memories (STM and LTM respectively). STM lasts from minutes to a few hours and is protein synthesis independent. In contrast, LTM could last from several hours to many years and involves changes in protein synthesis, gene expression and synaptic plasticity (Mayford et al., 2012; Dudai et al., 2015).

LTM is currently divided in at least three phases: acquisition, consolidation and retrieval. Memory consolidation is the process through a new memory is saved as a LTM suitable to be evoked under certain stimulus (see Lynch, 2004; Dudai et al., 2015). Spatial memories are mainly consolidated in the hippocampus (see Izquierdo et al., 1992), associative memories are related to different cortical areas (see Izquierdo et al., 1992; Gilmartin and Helmstetter, 2010; Reis et al., 2013; Kwapis et al., 2015), and memories with strong emotional components are mainly associated with the amygdala (see LeDoux, 2000; McGaugh et al., 2002). At the cellular level, memory consolidation involves new protein synthesis (see Jarome and Helmstetter, 2014) since the infusion of translation inhibitors (i.e., cycloheximide or anysomicin) during early memory consolidation impairs retrieval in several paradigms (Rossato et al., 2007; Artinian et al., 2008; Gilmartin and Helmstetter, 2010; Reis et al., 2013; Kwapis et al., 2015).

Hippocampal NMDA receptors (NMDAR) are required for memory acquisition and/or consolidation. Pretraining intrahippocampal perfusion of NMDAR antagonists in rodents cause spatial (Vianna et al., 2000) and contextual memory impairment (Cammarota et al., 2004; Schenberg and Oliveira, 2008). AP5 injection immediately after a 1 or 2 min open field (OF) session impaired LTM (tested 24 h later; Izquierdo et al., 1992), while the same AP5 dose given immediately after a 5 min session in the OF has no effect in LTM-habituation tested 24 h after training (Vianna et al., 2000).

NMDAR are heterotetramers composed by 2 GluN1 obligatory subunits and 2 GluN2 (A-D) or GluN3 (A-B) regulatory subunits. In central nervous system (CNS) regions involved in cognitive functions, like the hippocampus and prefrontal cortex (PFC), GluN2A and GluN2B are the major regulatory subunits (see Paoletti et al., 2013; Sanz-Clemente et al., 2013). The expression of these regulatory subunits is dynamic and tightly regulated (see Lau and Zukin, 2007; Yashiro and Philpot, 2008; Sanz-Clemente et al., 2013). GluN2B is the major regulatory subunit along embryonic development and hence, GluN2B containing NMDAR (GluN2B-NMDAR) are predominant during prenatal life, particularly in the hippocampus and PFC (Sans et al., 2000). Early after birth, there is an increase in GluN2A expression, both in transcription and translation, while GluN2B expression remains constant; as a consequence, GluN2A/GluN2B ratio raises up during that period (Monyer et al., 1994; Hoffmann et al., 2000). This increase in GluN2A/GluN2B ratio is known as the “NMDAR developmental switch” (see Yashiro and Philpot, 2008; Sanz-Clemente et al., 2013).

NMDAR pharmacological blockade or GluN1 knockdown affected long-term potentiation (LTP). AP5 treatment before or during plasticity induction caused LTP failure (Selig et al., 1999). Electrophysiology assays in *Grin1*^∆DGCA1^ mouse showed that LTP induction was preserved in CA3, while CA3-CA1 LTP was abolished, indicating that “normal NMDAR expression and distribution” was a necessary condition for LTP induction (Bannerman et al., 2012).

Knocking down GluN1 expression in rat hippocampus by an amplicon vector expressing a specific GluN1-antisense RNA, led to deficits in LTM memory of an inhibitory avoidance task and of habituation to an OF (Adrover et al., 2003; Cheli et al., 2006). Bannerman et al. (2012) reported that a CA1 and dentate gyrus (DG) GluN1 KO mouse (*Grin1*^∆DGCA1^) was able to form LTM of the spatial Morris water maze task, but the spatial reference memory was impaired in a radial maze task, suggesting that CA1 and DG NMDAR play an important function in using spatial knowledge to select between alternative responses that arise from competing or overlapping memories (Bannerman et al., 2012). On the other hand, mice lacking hippocampal either GluN2A or GluN2B subunit were able to acquire spatial memories (see Bannerman, 2009; Shipton and Paulsen, 2014). However, mice lacking hippocampal GluN2A signaling have impairments in spatial working memories (Bannerman et al., 2008). Also, as reviewed by Bannerman (2009), the lack of either GluN2A or GuN2B regulatory subunits or knocking down GluN1 (which means the entire receptor) in different brain regions and/or hippocampal sub-regions, led to different degrees of impairment in spatial memory performance.

Concerning the dynamics of NMDAR subunits after LTP induction, Barria and Malinow (2002) reported an increase in GluN2A-NMDAR at dendritic spines 30 min after LTP induction in hippocampal slices from neonatal rats (Barria and Malinow, 2002). Accordingly, Grosshans et al. (2002) showed that after plasticity induction, GluN1 and GluN2A subunits increased in the synaptic fraction, with a corresponding subunits decrease in non-synaptic fractions, in slices from 6 to 8 week old rats (Grosshans et al., 2002). Later on, Bellone and Nicoll (2007) reported that LTP induction in fresh hippocampal slices from newborn mice led to a rapid change (in milliseconds to seconds) from GluN2B-NMDAR to GluN2A-NMDAR mediated currents (Bellone and Nicoll, 2007). These results strongly suggest that there was an increase in synaptic GluN2A-NMDAR, which was attributed to mobilization of previously assembled NMDAR from non-synaptic pools. Other authors have investigated changes in NMDAR subunits *in vivo* at different time points after LTP induction. It was shown that there are several waves of increase in NMDAR subunits: i.e., GluN2A and GluN2B total levels in DG were increased 20 min and 48 h after LTP induction by high frequency stimulation (HFS; Williams et al., 1998), while GluN1 levels were increased 8 h and 48 h after HFS (Williams et al., 2003; Kennard et al., 2009).

NMDAR subunits increase was also reported to occur after memory acquisition. Subramaniyan et al. (2015) showed that there was an increase in GluN1 and GluN2A complexes in dorsal hippocampus synaptosomal fraction 6 h after training in a holeboard (along 3 days) and stimulated (before the third session) with a weak tetanizing stimulus (which *per se* was not able to lead to L-LTP). However, they have also shown that there was an increase in GluN1 and GluN2B hippocampal complexes in synapstosomal fraction extracted 6 h after 10 days training in a radial maze, without electric stimulation (Shanmugasundaram et al., 2015). In the same work, an increase of both GluN1 and GluN2A subunits was shown to occur at the same time in PFC complexes.

In a previous work, we have shown that following habituation of young adult rats to a new environment (a hippocampus dependent task) and after effective LTP induction by theta burst stimulation (TBS) in fresh hippocampal slices, there was an increase in hippocampal GluN1 and GluN2A subunits, about 70 min later. Therefore, this rise would take place during early memory consolidation phase and likely, during the establishment of an “effective” long-term synaptic plasticity (Baez et al., 2013).

In the present work, we have investigated the occurrence and timing of NMDAR subunits changes after habituation of young juvenile and adult rats to a new environment, to find out whether it is a general phenomenon along life. We have also investigated if similar changes occurs following an object recognition (OR) task in adult rats, in an attempt to further understand their putative relationship with learning and memory processing. In addition, we have studied NMDAR subunits increase after plasticity induction in primary cultures of hippocampal neurons, to further understand its dynamics and some of the involved mechanisms.

## Materials and Methods

### Behavioral Task

Male Wistar rats were acquired in the animal facility of the Facultad de Ciencias Exactas y Naturales (UBA), and then housed at the IBCN Animal Facility. Animals were maintained in groups of 4–5 per cage, under a 12-h light/dark inverted cycle (lights on: 8 p.m.), at 25°C ± 1 room temperature, with food and water *ad libitum*. All the procedures involving animals were carried out in accordance to the guidelines of the USA National Institutes of Health Guide for the Care and Use of Laboratory Animals and were approved by the Animal Care and Use Committee of the University of Buenos Aires (CICUAL, Facultad de Medicina, UBA).

#### Open Field (OF)

One, two and three month old male rats were exposed to an OF (75.0 cm long × 75.0 cm wide × 40.0 cm high) for 5 min (training session). This arena contained different visual clues on its walls and a grid designed in the floor (squares of 15.0 cm × 15.0 cm). Other animals were trained and then re-exposed for 5 min to the same OF 24 h later (test session). The number of crossings across the grid lines were recorded and compared to evaluate habituation to the environment. After OF training or test, rats were euthanized at different time points (as indicated for each group of assays in the “Results” Section); hippocampus, PFC and amygdala were dissected and homogenized for NMDAR subunits analysis by western blot (WB).

#### Object Recognition (OR)

Three month old adult rats were exposed to an OF 10 min per day in three consecutive days, as to induce habituation. On the 4th day, two similar objects (A-A′) were added to the OF. Then, rats were left to freely explore them during 5 min (training session, “object exposure phase”) and the time the animal spent exploring each object was recorded. After training, animals were euthanized either immediately or 70 min later. Other groups of rats were returned to their home cages after OR training. On the 5th day, these rats were exposed for 5 min to the same familiar objects (A-A′) or to a familiar and a new object (A-B; Test session). Discrimination index was calculated as time exploring right object − time exploring left object/time exploring right object + time exploring left object. Immediately or 70 min after test, animals were euthanized and the hippocampus and PFC were dissected, homogenized and prepared for WB analysis.

Objects used for this task were similar in textures and sizes (i.e., about 10 cm high and 8 cm wide), but with distinctive shapes (i.e., pyramids and hemispheres). Objects and positions were counterbalanced across experiments and behavioral trials.

### Western Blot (WB)

Hippocampus, PFC and amygdala from each animal exposed to OF or OR were homogenized separately in a Teflon glass potter (5 × 15″), in 100 mM NaCl, 0.2% Triton X-100, 1 mM EGTA, antiproteases cocktail (Sigma), 20 mM HEPES buffer (pH 7.4); and then incubated on ice 30 min to “complete tissue lysis”. To avoid overloading the gel, protein concentration was preliminary estimated using the BCA kit (Sigma) with highly diluted (>100 folds) aliquots. Samples were resuspended in Laemmli buffer and cracked at 100°C for 5 min. All samples were individually processed and analyzed. Protein samples were separated on a 10% SDS-PAGE gel and transferred to a polyvinylidenedifluoride membrane (Immobilon-P, Millipore). Blots were blocked with 3% non-fat milk-0.05% Tween-20 in Tris buffer saline (TTBS) and incubated with primary antibodies: anti-GluN1 (rabbit polyclonal 1:1000, Sigma), anti-GluN2A (rabbit polyclonal, 1:1000 Chemicon) or GluN2B (rabbit polyclonal, 1:1000 Chemicon); and anti-GAPDH (1:5000, Sigma). After wash-out, blots were incubated with HRP-conjugated anti-rabbit secondary antibody (1:2000; Amersham Biosciences, GE Healthcare, Piscataway, NJ, USA) or HRP-conjugated anti-mouse secondary antibody (1:5000; Sigma), developed in SuperSignal West Pico Chemiluminescent Substrate solution (Thermo Scientific, Waltham, MA, USA) and exposed to film (Agfa-Gevaert NV, Mortsel, Belgium).

To determine the actual amount of NMDAR subunits, the intensity of a band corresponding to each NMDAR subunit was relativized to the corresponding GAPDH band used as internal control, in each assay.

### Primary Neuron Cultures, Stimulation and Immunofluorescence

Hippocampal neuron cultures were performed as described by Kaech and Banker (2007), with some modifications. Briefly, both hippocampi were dissected from Wistar rat’s embryos (E17) and digested with trypsin (Sigma, Sigma-Aldrich Co, St. Louis, MO, USA). Cells were plated onto poly-L-lysine (Sigma)-coated glass coverslips (Waldemar Knittel Glasbearbeitungs—GmbH, Germany) and incubated with Neurobasal (NB; Invitrogen, Life Technologies Corporation, Carlsbad, CA, USA) supplemented with B27 (Invitrogen) and glutamine (Invitrogen; complete NB). Cultures were maintained in complete NB at 37°C and with 5% CO_2_. Culture media was replaced by halves every 3 days (Kaech and Banker, 2007).

Cell cultures of 18 days *in vitro* (DIVs) were stimulated with 55 mM KCl, as described in Wu et al. (2001). Immediately or at different time points after stimulation, cultures were fixed in paraformaldehyde 4%-sucrose 4% (Sigma)-phosphate buffer saline (PBS). Some cultures were fixed without any treatment and used as controls. When required, either Cycloheximide (CHX, 40 μg.ml), Actinomycin D (ActD, 40 μM) or PBS was added to the media 10 min before KCl stimulation and each drug was maintained there by appropriate replacement of conditioned media until cultures were fixed.

For immunofluorescence assays, coverslips were permeabilized in 0.1% Triton X-100-PBS. Then, coverslips were blocked with 5% normal sheep serum-0.05% Tween-20, PBS (TPBS). After blocking, cultures were incubated with anti-GluN1 (rabbit polyclonal, 1:100, Sigma) or anti-GluN2A (rabbit polyclonal, 1:100, Chemicon, Millipore, Billerica, MA, USA) antibodies. Coverslips were washed with PBST and then incubated with Cy2-conjugated anti-rabbit secondary antibody (1:500, Jackson ImmunoResearch Laboratories, Inc., West Grove, PA, USA). After washing with TPBS, coverslips were mounted using Fluorescence Mounting Medium (Dako, Agilent Technologies, Glostrup, Denmark).

### Image Analysis

Images from immunofluorescence assays were obtained under an Olympus-IX81 microscope and a FV300 Olympus Confocal microscope (Olympus CO, Tokyo, Japan). Images were analyzed with ImageJ software (ImageJ, NIH, Bethesda, MA, USA)^1^. Mean fluorescence was determined in 600× field images. Immunofluorescence of each neuronal body was estimated by ImageJ. To evaluate changes in NMDAR subunits at dendrites, 1000× images were used. *Puncta* were counted in isolated dendrites and were normalized to 10 μm. Each experiment was performed in duplicate in three independent cultures.

### Statistical Analysis

For WB and immunofluorescence, variables were first analyzed for normality by Kolmogorov Smirnov normality test. Then, analysis was performed either by Student’s *t* test (checking homoscedasticity by *F* test to compare variances) or by ONE WAY ANOVA (using Bartlett’s test to compare variances). ONE WAY ANOVA was followed by post-test analysis via Newman-Keuls or Dunnett tests, when appropriate. All these data are expressed as Mean ± SEM.

On the other hand, as behavioral parameters did not adjust to a Gaussian distribution, these data were analyzed using non-parametric statistic (Kruskal Wallis Test or Mann Whitney) and expressed as medians with interquartile ranges. For each set of experiments, the statistic test used is indicated in the figure legend. Data analysis was performed using the GraphPad Prism 6.0 program (GraphPad Software, Inc., San Diego, CA, USA).

## Results

### NMDAR Subunits Changes *in Vivo*, after OF Habituation

#### Hippocampal NMDAR Subunits Levels after Habituation at Different Ages

We have previously shown that hippocampal GluN1 and GluN2A NMDAR subunits were increased, whereas GluN2B appeared to remain unchanged, 60–70 min following either synaptic plasticity induction by TBS *in vitro*, in fresh hippocampal slices or after habituation to a new environment in adult Wistar rats (Baez et al., 2013).

**Figure 1:**
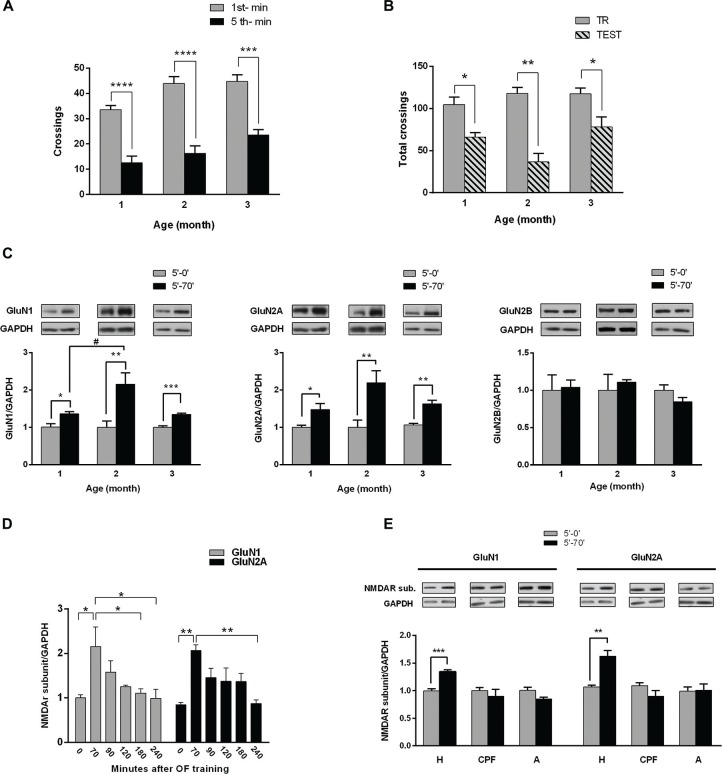
**Hippocampal NMDA receptors (NMDAR) subunits levels after habituation of rats to an open field (OF). (A)** Training session in an OF. One, two and three month (m) old male rats were exposed for 5 min to the arena; bars indicate number of crossings in the 1st (white) and 5th min (black). There is a significant decrease in number of crossings in the last minute compared with the 1st, at the three different ages (***p < 0.005, ****p < 0.0001 by Wilcoxon test; 1 m *n* = 8, 2 m *n* = 16, 3 m *n* = 12). **(B)** Total crossings performed by rats trained in the OF and tested in the same arena 24 h later. There were significant differences between total number of crossings in the test (gray bars) and in the training (striped bar) at the three ages (*p < 0.05, **p < 0.01. Wilcoxon test; *n* = 8 for all groups). **(C)** Hippocampal GluN1, GluN2A and GluN2B subunits levels after training. Rats were euthanized immediately or 70 min after training session (0′ post-TR, 70′ post-TR groups). Both GluN1 and GluN2A subunit levels increased 70 min after OF training session at the three ages (*p < 0.05, **p < 0.01, ***p < 0.001, unpaired *t*-test; ^#^*P* < 0.05 ONE WAY ANOVA, Newman-Keuls Multiple Comparison Post-Test; *n* = 5 for each subunit in 2 and 3 month old rats; while *n* = 4 for 1 month old rats). Insert on top: representative western blot (WB) bands for GluN1, GluN2A and GluN2B NMDAR subunits and GAPDH (used as internal control).** (D)** Temporal course of NMDAR subunits increase after OF training (*P < 0.05, ONE WAY ANOVA, Newman-Keuls Multiple Comparison Post-Test, *n* = 6 for each time group). **(E)** GluN1 and GluN2A levels at the hippocampus (H), prefrontal cortex (PFC) and amygdala (A), measured immediately or 70 min after OF training. GluN1 and GluN2A levels significantly changed only in the hippocampus 70 min after OF (**p < 0.01, ***p < 0.001. Unpaired *t* test).

To investigate if those changes would be a general feature following habituation to a new environment, we assessed NMDAR subunits in 1, 2 or 3 month old male Wistar rats, after exploration of an OF for 5 min (training session, Figure 1A), since it is long enough to induce habituation (Izquierdo et al., 1992; Baez et al., 2013). Exploration parameters progressively decreased from the 1st to the 5th min along the training session, at each age analyzed, indicating that STM of habituation to the OF was formed (Figure 1A). The number of crossings was not significantly different between the three ages. Some of those rats were euthanized to analyze NMDAR subunits (see next paragraph). In the following day (24 h later), some animals were left to explore the same arena for 5 min (test session) to evaluate LTM; total number of crossings was significantly lower during test than during training session, indicating that habituation to the OF was consolidated (Figure 1B).

We have assessed NMDAR subunits level in 1, 2 and 3 month old male rats following OF training (Figure 1C). Either immediately (5′-0′ group) or 70 min (5′-70′ group) after the training session, rats were euthanized and both hippocampi were dissected and processed for WB. The levels of both GluN1 and GluN2A subunits were significantly higher in 5′-70′ group than in 5′-0′ group, in hippocampal total protein extracts from 1, 2 and 3 month old rats (Figure 1C). GluN2B subunit level was not significantly different between 5′-0′ and 5′-70′ groups (Figure 1C). In addition, GluN2B levels were similar in the three ages (data not shown).

On the other hand, the increase in GluN1 was significantly different between 1 and 2 month old rats, though not between 2 and 3 month old animals; while the increase in GluN2A was not significantly different between the three ages analyzed.

#### *In Vivo* Time Course of Subunits Change

In order to find out how long GluN1 and GluN2A changes last *in vivo*, we analyzed hippocampal NMDAR subunits longer after OF habituation. Hence, some of the 3 month old rats trained in the OF for 5 min were euthanized at different intervals after OF session. WB analysis corroborated that hippocampal GluN1 and GluN2A levels were significantly increased 70 min after training, decreasing to levels not significantly different from controls 90 min after OF exploration. GluN1 level was significantly lower than the maximum peak at 180 min, though not at 90 or 120 min after training (Figure 1D). In addition, GluN2A level was not significantly lower than the maximum increase until 240 min after exploration/habituation to the OF (Figure 1D). These results showed that the increase in GluN1 and GluN2A subunits was transient and suggest that it could last longer than 90 min.

#### NMDAR Subunits Levels in Other Central Structures Related to Memory Processing

Hippocampus, amygdala and PFC are brain regions associated with memory acquisition and/or consolidation (McGaugh et al., 2002; Neves et al., 2008; Dudai et al., 2015). To find out if NMDAR subunits raise occurs in other related areas, we assessed GluN1 and GluN2A in protein extracts from hippocampus, PFC and amygdala, in animals euthanized immediately or 70 min after OF training. As shown in Figure 1E, GluN1 and GluN2A subunits level was significantly higher only in hippocampal samples from 5′-70′ group (34 ± 3% for GluN1 and 56 ± 10% for GluN2A, compared with 5′-0 group), while no significant differences were observed either in amygdala or in PFC.

Altogether, these results showed that GluN1 and GluN2A transiently increased after habituation to the OF and that those changes only occurred in the hippocampus, at least at the evaluated time points.

### NMDAR Subunits Level after Object Recognition Task (OR)

If the observed raise in NMDAR subunits would be related with memory consolidation, GluN1 and GluN2A increases should be expected to occur after other learning paradigms.

**Figure 2:**
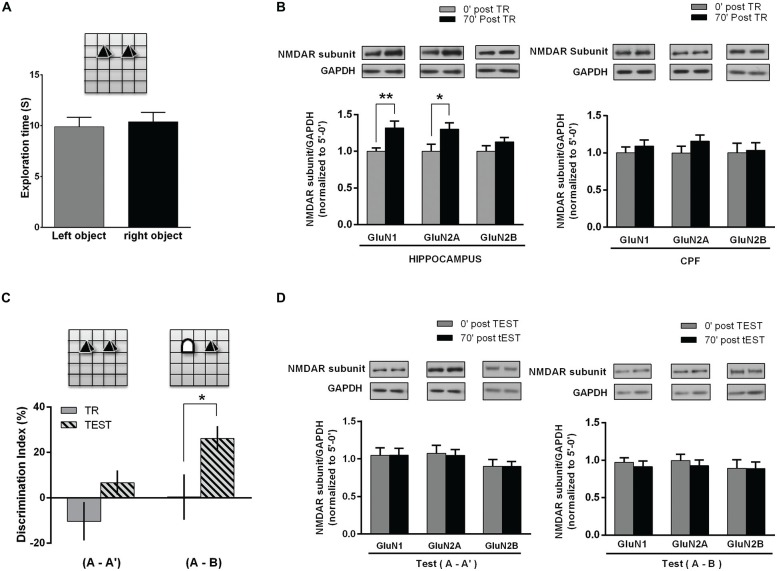
**NMDAR subunits in the hippocampus and PFC of rats following NOR task. (A)** Object exposure phase (Training session); rats were exposed to two identical objects for 5 min in and already familiar OF. There was no significant difference between exploration times of each one of two identical objects. **(B)** Hippocampus and PFC NMDAR subunits after training. Rats were euthanized immediately or 70 min after the task (0 post-TR, *n* = 10; 70′ post-TR, *n* = 10). WB analysis showed a significant increase in hippocampal GluN1 and GluN2A levels for 70′ post-TR group (*p < 0.05, **p < 0.01. Unpaired *t* test), while there was no significant change in PFC subunits level. Insert on top: representative WB bands for GluN1, GluN2A and GluN2B NMDAR subunits and GAPDH (used as internal control). **(C)** NOR test session. Rats were reintroduced in the arena 24 h after training and again exposed to two objects for 5 min, either a familiar and a novel object (**(A-B)** group *n* = 11) or two familiar objects (**(A-A′)** group *n* = 11). Rats explored significantly longer the novel object than the familiar one (*p < 0.05. Paired *t* test). **(D)** NMDAR subunits level in the hippocampus after NOR TEST. Some rats were euthanized immediately and others 70 min after TEST (0′ post-TEST *n* = 5; 70′ post-TEST *n* = 6); there were no significant differences in GluN1 and GluN2A levels after test, both in rats exposed either to two familiar objects **(A-A′)** or to a new and a familiar object (**A-B**; Unpaired *t* test). Insert on top: representative WB bands for GluN1, GluN2A and GluN2B NMDAR subunits and GAPDH (used as internal control).

Therefore, 3 month old male Wistar rats were trained in an OR task as follows: along three consecutive days each rat was left into the OF for 10 min/day. On the 4th day, two identical objects (A-A′) were placed in the arena and each rat was left to freely explore the objects for 5 min (training session, corresponding to the “object/s exposure phase”). Exploration time of each object was similar, denoting no preferences regardless object’s position (Figure 2A). Immediately or 70 min later, hippocampus and PFC of each animal were dissected and individually homogenized for NMDAR subunits WB assays. Both GluN1 and GluN2A subunits levels were significantly higher in hippocampal samples from animals euthanized 70 min after OR training, compared to those euthanized immediately after training. Instead, no significant changes were observed in PFC. GluN2B subunit level was not different from the respective controls in all the analyzed groups, either in the hippocampus or in the PFC (Figure 2B).

These results are in agreement with those observed after habituation to the OF and show that hippocampal GluN1 and GluN2A subunits significantly increase following a supposedly relevant modification of the environment, in spite of the previous habituation to the same arena.

As previously reported (Baez et al., 2013), hippocampal NMDAR subunits did not differ from controls after a 2nd OF session carried out 24 h after the 1st one. Taking into account that OR test could be performed with similar or different objects, including a new one, and thus, “changing the environment” (experience), we decided to assess NMDAR subunits in another group of rats, after testing them 24 h later. During the object exposure phase (or training) performed on the 4th day, animals explored the similar objects (A-A′) without preferences. On the 5th day, rats were separated in two different groups: (1) rats exposed to the same (familiar) objects than in the training session (A-A′); and (2) rats exposed to a familiar and a new object, by replacing one of the familiar objects (A-B). As expected, during test session rats exposed to (A-A′) spent a similar time exploring each object, while rats exposed to a familiar and a new object (A-B) spent significantly longer exploring the new object, indicating that a memory of the old object and of the previous spatial configuration, which would allow discrimination, was already consolidated (Figure 2C). After the test, rats were euthanized immediately or 70 min later. Then, hippocampus and PFC were dissected and NMDAR subunits were analyzed by WB. As it is shown in Figure 2D, there were no changes in GluN1, GluN2A and GluN2B subunits 70 min after test, either in rats exposed to the familiar objects or in those animals exposed to a new and a familiar object (Figure 2D).

These results show that there is an increase in GluN1 and GluN2A subunits in the hippocampus following the object exposure phase in OR task, though not after test, even when one object was effectively discriminated as a new one.

### Dynamics of NMDAR Subunits Change after Plasticity Induction

#### Time Course of the Rise in GluN1 and GluN2A Subunits

A rise in GluN2A dendritic expression was reported in primary cultured neurons immediately after plasticity induction (Udagawa et al., 2012; Swanger et al., 2013). This increase continued up to 30 min post-stimulation, and was mainly attributed to local translation.

We have found that there was an increase of both GluN2A and GluN1 subunits about 1 h after synaptic plasticity induction and establishment, in electrophysiological assays carried out in rat hippocampal slices and in hippocampal neuron cultures stimulated with KCl pulses (Baez et al., 2013).

**Figure 3:**
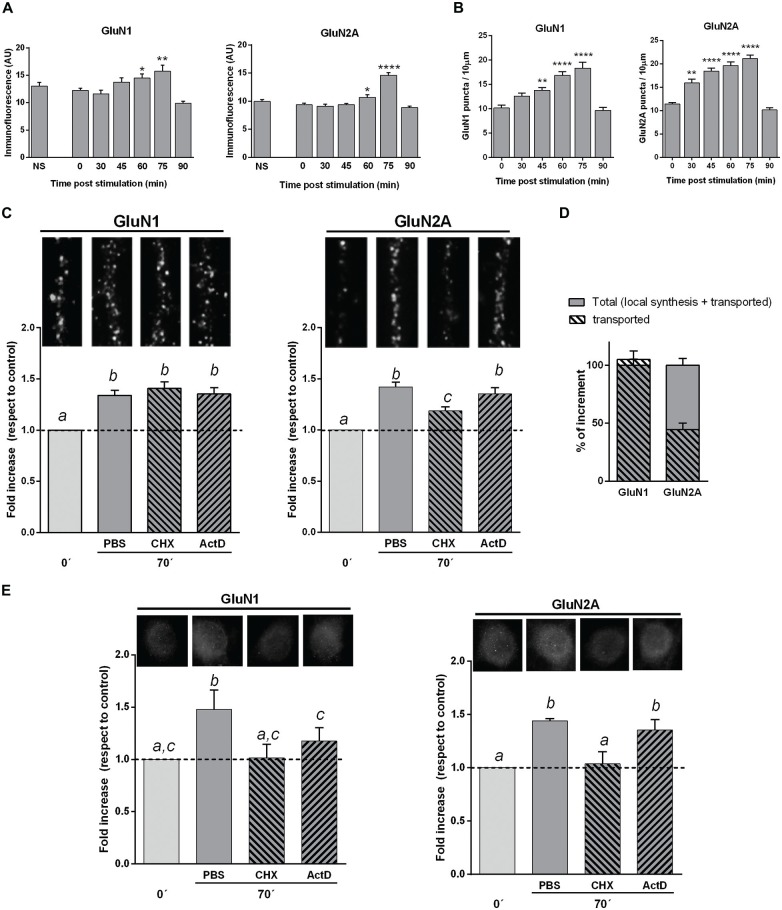
**NMDAR subunits in hippocampal mature neuron cultures stimulated by KCl pulses. (A)** GluN1 and GluN2A mean fluorescence of neuronal bodies at different times after KCl stimulation (Bars, Mean ± SEM). There was a significant increase in GluN1 and GluN2A mean fluorescence 60 and 75 min after KCl stimulation (*p < 0.05, **p < 0.005, ****p < 0.0001; ONE WAY ANOVA, Dunnett Post-Test, *n* = 50 for each time). **(B)** NMDAR subunits dendritic *puncta* (IF) at different times after KCl stimulation (same cultures than in **A**). There was a significant increase in both GluN1 and GluN2A *puncta* 45, 60 and 75 min after stimulation, while GluN2A was also significantly higher at 30 min, compared with cultures fixed immediately after stimulation (**p < 0.001, ****p < 0.0001; ONE WAY ANOVA, Dunnett Post-Test; *n* = 20 dendrites for each time). **(C)** Changes in dendritic NMDAR subunits *puncta* after blocking either transcription (Actinomycin D, ActD) or translation (Cycloheximide, CHX). Primary cultures were treated with CHX or ActD from 10 min before KCl stimulation. The increase in dendritic GluN1 *puncta* 75 min after stimulation was not affected by either CHX or ActD. CHX pretreatment partially blocked dendritic GluN2A *puncta* increase, while ActD had not effect. On top of each bar, it is shown a 10 μm long representative dendrite immunostained for each subunit (different letters, statistically significant differences; similar letters, non-significant differences between treatments; ONE WAY ANOVA, Tukey Post Test, *n* = 40 dendrites/treatment).** (D)** Mechanism of *in vitro* NMDAR subunits increase. Plain gray bars indicate total NMDAR subunits rise, as 100% (left: GluN1; right: GluN2A). Stripped bars represent the remaining dendritic subunit increase after blocking translation. GluN1 *puncta* increase mainly depends on NMDAR mobilization, since stripped and plain bars almost fully overlap. Rise in GluN2A dots depends on both local translation (55.6 ± 7%) and NMDAR mobilization (44.4 ± 5%).** (E)** GluN1 and GluN2A mean fluorescence of neuronal bodies in cultures pretreated with CHX or ActD (same cultures as in **C**). CHX fully blocked rise in both GluN1 and GluN2A. ActD partially blocked GluN1 increase, whereas it did not significantly affect GluN2A increase (Different letters, statistically significant differences; similar letters, non-significant differences between treatments; ONE WAY ANOVA, Tukey Post Test, *n* = 100 bodies/treatment).

To investigate the dynamics of these changes, we have used hippocampal neuron cultures (18 DIVs) stimulated by KCl pulses. At different intervals after stimulation, neurons were fixed and immunostained for GluN1 or GluN2A. To follow the time course of the subunits increase, we have measured the mean fluorescence for each subunit in the neuronal body. GluN1 and GluN2A mean fluorescence expressed in arbitrary units (U.A.), were similar to controls at 30 as well as at 45 min post-stimulation; then, at 60 and 75 min GluN1 and GluN2A levels were significantly higher than at 0, 30 and 45 min. On the other hand, 90 min after stimulation GluN1 and GluN2A levels were not significantly different from cultures either non-treated or fixed immediately after stimulus (Figure 3A). We have also analyzed GluN1 and GluN2A *puncta* in dendrites after plasticity induction. The density in GluN2A *puncta* was higher than controls when neurons were fixed 30, 45, 60 or 75 min after KCl pulses (Figure 3B). At variance, the density in GluN1 *puncta* was significantly higher than controls 45, 60 and 75 min after stimulation, though not at 30 min. Furthermore, both GluN1 and GluN2A *puncta* were not significantly different from controls 90 min after stimulus (Figure 3B), as it happened in neuronal bodies.

Therefore, GluN1 and GluN2A subunits increased at both dendrites and neuronal bodies; this rise was transient, reaching a maximum at 75 min and returning to control levels 90 min post-stimulus, reminding our results obtained after memory acquisition.

#### Mechanisms of the Rise in NMDAR Subunits

As it was mentioned in the “Introduction” Section, the increase in dendritic GluN2A *puncta* of cultured neurons during the first 30 min after stimulus, was mainly attributed to local translation of silent pre-localized mRNAs (Udagawa et al., 2012; Swanger et al., 2013).

To investigate which is the contribution of *de novo* translation to the rise in NMDAR subunits here reported, we have treated mature primary cultures of hippocampal neurons either with CHX (to block translation) or ActD (to block transcription), from 10 min before KCl stimulation up to the cells fixation (Figures 3C–E).

The density of GluN1 *puncta* in dendrites of KCl stimulated neurons either treated with CHX or ActD, were not significantly different than controls (Figures 3C,D). Thus, the increase in dendritic GluN1 *puncta* at 75 min appeared to mainly depend on already translated subunits that were mobilized by stimulus/activity. On the other hand, the rise in GluN2A *puncta* significantly decreased in dendrites when KCl stimulated neurons were treated with CHX, though it still remained significantly higher than controls (Figure 3C). This rise in GluN2A *puncta* was not significantly reduced by ActD, indicating that such increase in GluN2A was not dependent on transcription. These results indicate that the increase in GluN2A depends, at least in part, on translation of pre-existing mRNAs, in accordance with previous reports (Baez et al., 2013; Swanger et al., 2013). Altogether, our results suggest that the rise of GluN2A in dendrites would be due to both local translation (55.6 ± 7%) and transport from extra-synaptic and/or non-synaptic pools (44.4 ± 5%), while the increase in GluN1 *puncta* would mainly be dependent on its mobilization from other pools (Figure 3D).

As protein expression is compartmentalized in neurons, we have also estimated GluN1 and GluN2A mean fluorescence of neuronal bodies after treatment with either CHX or ActD (Figure 3E). CHX added to the media from 10 min before KCl stimulation completely blocked the increase in both GluN1 and GluN2A subunits, strongly suggesting that an active translation of both subunits would be involved. The rise in GluN1 was only partially blocked by ActD, suggesting that transcription could also be involved. On the other hand, the rise in GluN2A did not significantly change with ActD treatment (Figure 3E).

These results indicate that after plasticity induction *in vitro*, there is a transient increase in GluN1 and GluN2A subunits both in neuronal bodies and dendrites that would depend on different subcellular mechanisms, and suggest that new assembled GluN2A-NMDAR could be expressed in dendrites.

## Discussion

### NMDAR Subunits Change Following Habituation to the OF and Training in OR

It is widely accepted that NMDAR are required for memory acquisition and/or consolidation and that hippocampal NMDAR are particularly relevant for spatial memory. The lack of either GluN2A or GuN2B regulatory subunits or the knock-down of GluN1, which means the entire receptor, in different brain regions and/or hippocampal sub-regions, led to different degrees of impairment in spatial memory performance (see Bannerman, 2009). Bannerman et al. (2012) reported that a CA1 and DG GluN1-KO mouse (*Grin1*^∆DGCA1^) was able to form LTM of the spatial Morris water maze; but its spatial reference memory in a radial maze was impaired, suggesting that CA1 and DG NMDAR could play an important function in using spatial knowledge to select between alternative responses that arise from competing or overlapping memories (Bannerman et al., 2012). Using a CA1 or a CA3 GluN1-KO mouse, Place et al. (2012) showed that NMDAR in CA1 region selectively contributed to the spatial aspect of episodic memory, while NMDAR in CA3 were not essential (Place et al., 2012).

It was shown that mice lacking either GluN2A or GluN2B regulatory subunits in the hippocampus or adjacent regions were able to acquire spatial memories (see Bannerman, 2009; Shipton and Paulsen, 2014). However, spatial working memory was impaired in mice lacking GluN2A signaling (Bannerman et al., 2008).

We have previously shown that both GluN1 and GluN2A subunits increased *in vivo* following habituation of rats to a new environment (Baez et al., 2013). It was also shown that synaptic NMDAR are increased 6 h after the last of 10 day training in a radial maze (Shanmugasundaram et al., 2015) or after 3 days training in the holeboard (Subramaniyan et al., 2015).

As mentioned in the “Introduction” Section, GluN2A-NMDAR increased at the neuron surface 30 min after synaptic plasticity induction by HFS, in hippocampal slices of neonatal rats (Barria and Malinow, 2002; Bellone and Nicoll, 2007), in hippocampal slices and synaptosomal fractions of 4–6 week old (Yin et al., 2011, 2012) and 6–8 week old rats (Grosshans et al., 2002).

Interestingly, Williams et al. (1998) showed that there are several waves of NMDAR subunits increase after LTP induction by HFS in the rat DG; i.e., there was an increase in GluN2A and GluN2B levels 20 min and 48 h after LTP induction. At the same time points, there was an increase of GluN1 and GluN2B subunits in DG synaptosomal fraction (Williams et al., 2003). On the other hand, these subunits were not significantly different from controls 1 h or 4 h after stimulation (Williams et al., 1998), but GluN1 subunit was higher than controls 8 h and 48 h after HFS. Kennard et al. (2009) have shown that the GluN1 increment in synaptosomes 48 h after LTP induction was due to an increase of NMDAR at the surface fraction, though not in the post synaptic density fraction (Kennard et al., 2009).

In this work, we have corroborated previous results in 2 month old rats and found that in both younger (1 month old) and older (3 months old) rats, which were able to habituate to a new environment (OF) after a 5 min session, hippocampal GluN1 and GluN2A subunits levels were significantly higher at 70–75 min, than in those animals euthanized immediately after the OF session, while GluN2B level remained unchanged (Figure 1C). These results suggest that the observed rise in subunits is a general feature along life, at least for young juvenile and adult rats.

We have also investigated the time course and amplitude of such changes *in vivo*. GluN1 and GluN2A reached a maximum at about 70 min after OF habituation. Thereafter, the subunits levels fell down, being not significantly different from controls 90 min after training. These results show that the rise in GluN1 and GluN2A is transient (lasting 90 min or less), suggesting the possibility that it could be playing a physiological role in memory in spite of its relatively short duration. The time course of these changes overlaps with the early steps of memory consolidation, suggesting a possible relationship between them.

As it was reviewed in Jarome and Helmstetter (2014), both protein translation and proteasome activity are necessary for memory consolidation (Jarome and Helmstetter, 2014). Therefore, we can speculate that GluN1 and GluN2A would be translated and assembled as new NMDAR, then expressed in the synapse, changing the probability for plasticity during and after acquisition. Therefore, that increase might facilitate synaptic plasticity contributing to memory formation. This increase in NMDAR expression could be acting as a check point or as a synaptic labeling in memory consolidation. Then, that increment could be actively degraded by ubiquitination and proteasome activity, returning the system to a more stable state.

We have assessed NMDAR subunits in other central structures involved in memory acquisition and storage, as PFC and amygdala. No significant changes were found in these structures 70 min after habituation to the OF in one session. Henderson et al. (2012) have shown that there was an increase in same NMDAR subunits in the motor cortex of mice, after training them in a single pellet-reaching task. Therefore, we could not discard that similar changes could happen in other central structures and/or in other time intervals. More recently, it has been reported that 6 h after training rats in the radial maze (Shanmugasundaram et al., 2015) or in a hole-board (Subramaniyan et al., 2015), there was an increase in GluN1 and GluN2A in the hippocampal synaptosomal fraction. After training in the hole-board, GluN2B was unchanged, but it was increased in PFC synaptosomal fraction following training in the radial maze task. These data strengthen the idea that changes in different NMDARs -or in the subunits- during memory consolidation, seem to occur in several waves. However, changes in different subunits in different structures, with a different timing could also be attributable to the tasks used and the areas involved in specific memories.

We have already reported that there were no changes in GluN1, GluN2A and GluN2B hippocampal subunits level of rats exposed 1 min to the OF and euthanized 70 min later (Baez et al., 2013), indicating that the rise in rat hippocampal subunits would not be just due to exposure to novelty. Instead, subunits changes occurred whenever habituation to the environment has taken place. In addition, there were not significant changes in NMDAR subunits when rats explored twice the OF for 5 min, confirming the selectivity of that change after a unique training session in the OF and strongly suggesting that habituation rather than just exploration or locomotor activity, would be related to NMDAR subunits raise. On the other hand, a STM was formed during the OF session, which was followed by the GluN2A rise; hence, this appears to be in line with the relevant finding that the GluN2A^−/−^ mice exhibited STM deficits in spatial working memory (Bannerman et al., 2008), indicating that GluN2A-NMDAR are required for STM.

Several molecular changes were reported as putative molecular consolidation markers (Bousiges et al., 2010; Katche et al., 2013). Taking into account that different authors—including ourselves have reported changes in NMDAR subunits level, particularly in synapse or synaptic fractions, either involving or not *de novo* protein expression, following different stimuli and experiences (Williams et al., 1998, 2003; Barria and Malinow, 2002; Grosshans et al., 2002; Kennard et al., 2009; Henderson et al., 2012; Udagawa et al., 2012; Baez et al., 2013; Swanger et al., 2013), we propose that the increase in GluN1 and GluN2A here shown could be a hallmark for “spatial” memory consolidation. Hence, it is expectable that similar changes also happen after other learning paradigms with spatial content. Barker and Warburton (2011) have shown that recognition is associated to medial PFC, which is required for both long-term and short-term recognition memory for places, or for associations between objects and places (Warburton et al., 2013). Consequently, in this work we have examined GluN1 and GluN2A levels in the hippocampus and PFC after an OR task. In accordance with our findings on GluN1 and GluN2A rise following habituation to the OF, these subunits also increased in the hippocampus 70 min after OR training, corroborating our prediction for a similar subunits rise in this structure following training in another task. However, no significant differences were found in PFC subunits after either OR training or test, though NMDAR changes in PFC could not be discarded to occur at different time intervals. These results are in accordance with the idea that NMDAR subunits change here reported would be related to spatial learning/memory and hence, this could be seen only in the hippocampus, where a representation/map of the environment is being formed, likely leading to STM and LTM. In addition, it must be taken into account that those rats had explored three times the same OF in three consecutive days and the only difference in the 4th day was just the presence of two (similar) objects that were explored for 5 min.

Therefore, we suggest that an enough relevant environmental change would elicit significant changes in “recognition/habituation representation”, leading to changes in hippocampal NMDAR subunits. As expected, there was no increase in subunits level following a test session with two familiar objects, as happened after the OF test. Surprisingly, GluN1 and GluN2A levels appeared to remain unchanged after replacing a familiar object by a new one in the 5th day, in spite of an appropriate discrimination of the new object. Interestingly, mice without or reduced GluN2A expression showed learning impairments limited to STM and to the rapid acquisition of spatial information (see Shipton and Paulsen, 2014). Precisely, rapid acquisition is required to learn something in just one short training session, as it is the case in the tasks used in this work. However, if changes in NMDAR subunits were related to STM, a similar increase would be expected after OR session with the inclusion of a novel object. Hence, we can speculate that modifying the environment by just one object, while keeping the same arena with two objects of similar size, in similar positions as in the training session, would be just too slight modification to generate a fully new engram. Even when the animal was able to discriminate the new object and to form an appropriate discrimination memory, it is likely that the change of hippocampal NMDAR subunits only takes place during consolidation of a novel representation, though not during a slight modification of an already consolidated memory, at least in that structure.

Taking together, our *in vivo* results are in agreement with previously reported NMDAR changes after habituation and plasticity induction in rats and in other animal models, and indicate that: (1) the observed raise in hippocampal subunits 70 min after training seems to be structure-specific, at least within that time interval; (2) these changes are conserved in young juvenile and in adult rats; (3) they happen after OF habituation and after training in an OR task, strongly suggesting that this would be a general feature during early (spatial) memory consolidation; (4) although a relationship with STM should be further investigated, it seems to be related to early consolidation of new spatial information leading to LTM.

### NMDAR Subunits Increase after Plasticity Induction *in Vitro*

NMDAR participate in physiological plasticity in the CNS during development, as well as in synaptogenesis and synaptic plasticity along the whole life. NMDAR pharmacological blockade or GluN1 knockdown affected LTP. AP5 treatment before or during plasticity induction caused LTP failure (Selig et al., 1999). Electrophysiology assays in *Grin1*^∆DGCA1^ mouse showed that LTP induction was preserved in CA3, while CA3-CA1 LTP was abolished, indicating that normal NMDAR expression and distribution was necessary for LTP induction (Bannerman et al., 2012).

As referred in the “Introduction” Section, it is already known that synaptic and dendritic GluN2A-NMDAR increase immediately after plasticity induction *in vitro* (Bellone and Nicoll, 2007; Udagawa et al., 2012; Swanger et al., 2013). This rise in synaptic NMDAR, that lasts seconds to minutes following stimulation, was attributed to lateral mobility of previously assembled GluN2A-NMDAR (Bellone and Nicoll, 2007). The increase of synaptic GluN2A-NMDAR was followed up by several investigators from immediately to 30 min after plasticity induction (Barria and Malinow, 2002; Grosshans et al., 2002). Udagawa et al. (2012) and Swanger et al. (2013) have shown that GluN2A-NMDAR increase at 30 min was mainly due to GluN2A local translation from silent GluN2A mRNAs located in the spines, which were translated immediately after plasticity induction. Therefore, it was suggested that new NMDAR would be assembled using recently translated GluN2A subunits and GluN1 subunits translated before stimulation and retained inside the ER (Udagawa et al., 2012; Swanger et al., 2013). At the same time, we reported that both GluN1 and GluN2A subunits increase in mature cultures of hippocampal neurons after stimulation by KCl and also in hippocampal slices after LTP induction by TBS, within a rather similar time course (Baez et al., 2013).

In this work, we have corroborated that there is an increase in GluN1 and GluN2A subunits level after plasticity induction in mature primary cultures of hippocampal neurons. This increase appears to reach a maximum at 75 min after stimulation. Then, it decreased, being similar to controls at about 90 min after stimulation.

The timing pattern of IF for each subunit showed some differences in neuronal bodies and dendrites, being more similar for different subunits in the same compartment than for the same subunit in different compartments, suggesting a close related regulation between them (see Figure 3). GluN1 as well as GluN2A mean fluorescence in neuronal bodies increase 60 min after stimulation (Figure 3A). On the other hand, the rise of GluN1 and GluN2A *puncta* in dendrites, starting at 30–45 min, seems to be more gradual and compatible with other author’s previous reports (Udagawa et al., 2012; Swanger et al., 2013).

These *in vitro* results are in accordance with those obtained *in vivo* after habituation to the OF and following training in an OR paradigm, when GluN1 and GluN2A subunits also peak in the hippocampus at about 70 min and decrease at 90 min (Figure 1D), without significant changes in GluN2B. As the half-life time of synaptic proteins under basal conditions (see Hanus and Schuman, 2013) is ordinarily longer than the intervals in which “activity related” changes in subunits takes place in our assays, both *in vitro* and *in vivo*, we guess that changes in NMDAR subunits synthesis and degradation would play an important role, putatively driven by activity. Thus, it is feasible that after KCl stimulation, as after memory acquisition (GluN2A containing) NMDAR were actively recruited to synapses, setting neurons into a more plastic state. NMDAR subunits expression would be enhanced for a time interval enough long to lead to synaptic plasticity facilitation or establishment. Thereafter, NMDAR expression would return to basal levels and also, there could be an increase in degradation by proteasome targeting, in an activity dependent way. Kato et al. (2005) demonstrated that activity-dependent changes in neuronal NMDAR levels involve retrotranslocation and degradation by Fbx-2 ubiquitination-proteasome pathway. They suggested that activity would release synaptic GluN1, which is relocated in dendrites where it is ubiquitinated through Fbx-2, leading to GluN1 degradation by cytosolic proteasome (Kato et al., 2005). Nelson et al. (2006) reported that a co-chaperone/ubiquitin ligase (C-terminus of Hsc70-interacting protein) facilitates the ubiquitination and degradation of Fbx2-bound glycoproteins, including GluN2A subunit. Recently, as reviewed in Lussier et al. (2015), it was confirmed that GluN2A and GluN2B have several putative ubiquitination sites.

Concerning possible mechanisms involved in the rise of NMDAR subunits, we have shown that the increase in GluN1 subunit in neuronal bodies was fully reduced by CHX. This result reveals the involvement of protein synthesis in that rise. ActD also produced a partial reduction in that increase, indicating that transcription of new mRNAs was also involved. Surprisingly, the increase of GluN1 dendritic *puncta* following stimulation was not reduced by either ActD or CHX, suggesting that this rise would be mainly due to NMDAR mobilization from other pools. This leads to wonder what function the increase serves to. The differences in neuron bodies and dendrites respect to GluN1 are difficult to interpret and deserve further investigation. Although we do not know what is the meaning of that GluN1 *de novo* synthesis in neuronal bodies, we can speculate that: (1) it is feasible that the GluN1 subunit expression would not be fully inhibited by CHX (added 10 min before stimulation) and hence, such remaining *de novo* translation could be responsible for part of the dendritic rise in GluN1; or (2) the *in vitro* system would not be able to effectively use *de novo* synthesized GluN1. Therefore, in this last case the rise of GluN1 in dendrites would not include *de novo* translated subunits.

In addition, the time course of the increase in dendrites appears to be slightly different compared with bodies (Figures 3A,B).

On the other hand, GluN2A dendritic increase *in vitro* appears to depend on both GluN2A-mRNA translation and mobilization of preexisting GluN2A-NMDAR from other pools, in quite similar proportions. It would be feasible that lateral mobilization of pre-localized NMDAR along the membrane towards the synapse undergoing plasticity induction, would contribute to the rapid former steps of synaptic plasticity. As more GluN2A-NMDAR are required, local translation would provide GluN2A subunits for local assembly of receptors, what would be rapidly available. This explanation let us to speculate that distinct check points at synapse maturation could be differentially regulated, allowing to reach full maturation only to those synapses involved in—or closer to—that putative “plasticity induction zone”, while other synapses would remain in pre-mature stages. It also seems feasible that *in vivo*, these changes participate in hippocampal synaptic plasticity as part of the (spatial) memory trace, contributing to memory consolidation.

## Concluding Remarks

In this work, we have analyzed changes in NMDAR subunits levels *in vivo*, after habituation of rats to a new arena or after training in OR, and *in vitro*, in cultures of hippocampal neurons after plasticity induction. GluN1 and GluN2A subunits are increased in the hippocampus of young juvenile and adult rats 70–75 min after habituation to an OF or following training in an OR paradigm, strongly suggesting that this would be a general feature during early memory consolidation. These increases occur in the hippocampus but not in other CNS related structures. Thereafter, GluN1 and GluN2A subunits decreased, reaching control levels 90 min after training. Changes in the same subunits and within a rather similar time course were observed *in vitro*, in primary cultures of hippocampal neurons stimulated by KCl pulses to induce plasticity. In dendrites, the increase in GluN1 could be attributed to mobilization from other pools, while GluN2A rise could be due to both translation and mobilization in similar proportions. On the other hand, GluN2A subunit increase in neuronal bodies would mainly depend on translation, while GluN1 increase depends on both transcription and translation. Altogether, these results allow us to hypothesize that, after plasticity induction, there is an increase in GluN2A-NMDAR in dendrites that could act as a signal contributing to label synapses involved in the plasticity induced by that particular activity, i.e., stabilizing and/or tagging them, facilitating the establishment of synaptic plasticity. We hypothesize that these changes *in vivo* would contribute to synaptic plasticity as part of the (spatial) memory trace in the hippocampus.

## Author Contributions

MCC performed behavioral and westernblot essays. She also, assisted in neuronal cultures. MCC analyzed statistically all experiments in this manuscript and collaborated with manuscript redaction. CAV participated in behavioral essays, helped in westerblot essays and immunofluorescences; also analyzed dendritic NMDAR puncta. EK, AIA, NC collaborated in behavioral essays. EK helped with statistical analysis. MS helped with behavioral and statistical analysis. MVB performed neuronal cultures, immunofluorescence and microscope analysis and also, MVB helped in statistical analysis. DAJ and MVB planned and organized experiments; coordinated the group and wrote the manuscript.

## Funding

This research was funded by Agencia Nacional de Promoción Científica y Tecnológica (ANPCyT; PICT-2013-2221); Universidad de Buenos Aires (UBA; UBACyT 20020130100575BA); Consejo Nacional de Investigaciones Científicas y Técnicas (CONICET; PIP 00975). MCC and AIA have fellowships from CONICET; CAV, NC and MS have fellowships from UBA.

## Conflict of Interest Statement

The authors declare that the research was conducted in the absence of any commercial or financial relationships that could be construed as a potential conflict of interest.

## Footnotes

^1^http://imagej.nih.gov/ij/
